# Integrated EWM-RSM optimization of wine-steaming enhances the anti-inflammatory activity of *Dendrobium huoshanense* polysaccharides

**DOI:** 10.3389/fchem.2026.1789581

**Published:** 2026-06-01

**Authors:** Wenli Jiang, Zhengfeng Wang, Mengjuan Ye, Jiahui Lv, Mengzhen Ma, Daiyin Peng, Lan Han, Weiyi Feng, Qiaosong Li, Nianjun Yu, Lihua Xing

**Affiliations:** 1 College of Pharmacy, Anhui University of Chinese Medicine, Hefei, Anhui, China; 2 Institute of Traditional Chinese Medicine Resources Protection and Development, Anhui Academy of Chinese Medicine, Hefei, Anhui, China; 3 MOE-Anhui Joint Collaborative Innovation Center for Quality Improvement of Anhui Clinical Chinese Medicinal Materials, Hefei, Anhui, China; 4 Anhui Province Key Laboratory of R&D of Chinese Medicine, Anhui Province Key Laboratory of Chinese Medicinal Formulae, Hefei, Anhui, China; 5 Anhui Yipin Mountain Villa Modern Agricultural Development Co., Ltd., Qianshan, Anhui, China

**Keywords:** anti-inflammatory activity, *Dendrobium huoshanense*, polysaccharides, processing technology, wine-steaming

## Abstract

**Background:**

*Dendrobium huoshanense* is a valuable medicinal herb in Traditional Chinese Medicine renowned for its polysaccharides which are considered a core pharmacological component, particularly for their anti-inflammatory properties. The herb is commonly subjected to wine-steaming processing to enhance its efficacy, yet the scientific rationale behind this traditional method remains insufficiently explored.

**Methods:**

This study systematically optimized the wine-steaming processing parameters for *D. huoshanense* and comparatively evaluated the anti-inflammatory activities of its polysaccharides before and after processing. The physicochemical properties of the polysaccharides before and after wine steaming were characterized by scanning electron microscopy (SEM), size exclusion chromatography coupled with multi-angle light scattering and refractive index detection (SEC-MALS-RI), and high-performance liquid chromatography The anti-inflammatory effects were assessed using lipopolysaccharide (LPS)-stimulated RAW264.7 macrophages. Key evaluations included the measurement of nitric oxide (NO) production and the analysis of mRNA expression levels of pro-inflammatory cytokines, namely TNF-α, IL-1β, IL-6, IL-10, iNOS, and COX-2.

**Results:**

The optimal wine-steaming conditions were determined as follows: moistening with 38% yellow rice wine for 7.5 h followed by steaming for 5 h. Following processing, the polysaccharide yield slightly decreased from 27.0% ± 0.5% in the raw sample to 26.0% ± 0.4%. a significant reduction in molecular weight, and altered monosaccharide composition with a decreased mannose-to-glucose ratio. *In vitro*, the wine-steamed polysaccharides exhibited stronger anti-inflammatory activity than those from the raw herb, with the most pronounced effect observed at 200 μg/mL, as reflected by greater inhibition of NO production and stronger downregulation of TNF-α, IL-1β, IL-6, IL-10, iNOS, and COX-2 mRNA expression.

**Conclusion:**

Wine-steaming processing significantly enhances the anti-inflammatory activity of *D. huoshanense* polysaccharides. These findings provide modern pharmacological evidence supporting the traditional processing method and offer a scientific basis for the standardized processing of this medicinal herb and the development of its polysaccharide-based active ingredients.

## Graphical Abstract

**Figure:**
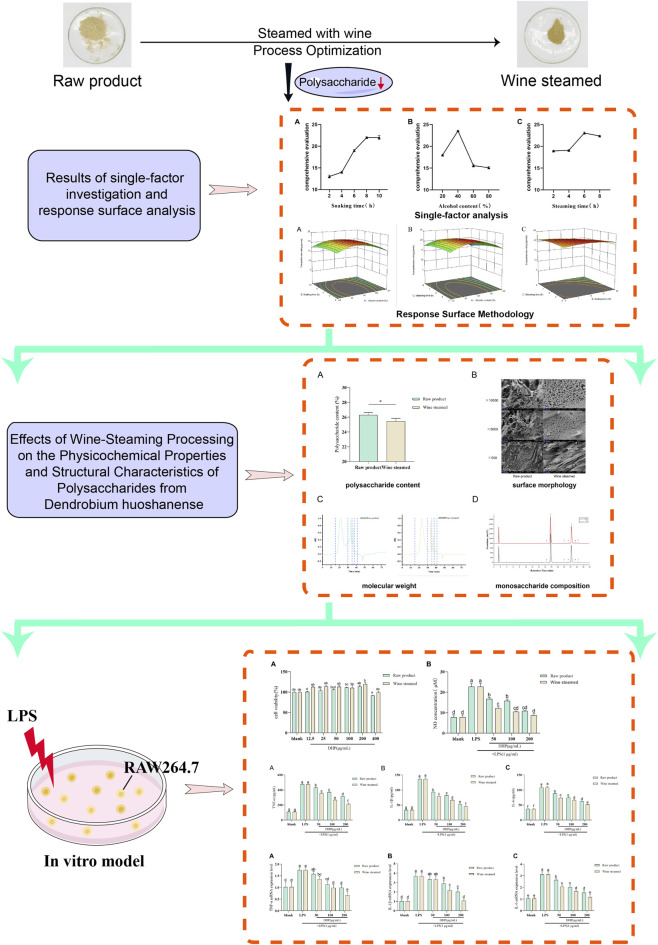

## 1 Introduction

*Dendrobium huoshanense* (*D huoshanense*), an endangered and endemic medicinal orchid of China, holds a distinguished position within the *Dendrobium* genus (Orchidaceae). Thriving in specific micro-environments, this rare plant has been historically treasured in Traditional Chinese Medicine (TCM) for its purported benefits in alleviating arthralgia, regulating Qi, improving gastrointestinal function, and promoting longevity, as documented in classical texts such as the *Shennong Ben Cao Jing* (Gao et al., 2022). Modern phytochemical studies have confirmed that it is rich in bioactive components, including polysaccharides, amino acids, bibenzyls, and flavonoids, which collectively contribute to a wide spectrum of pharmacological activities such as anti-inflammatory, anti-tumor, hypoglycemic, and hepatoprotective effects (Li et al., 2015; Shang et al., 2021). Among these, polysaccharides are considered one of its core pharmacologically active components.

The processing of crude medicinal materials, known as Paozhi, is a cornerstone of TCM practice. Techniques such as steaming, stir-frying, or wine-processing are employed to modify the properties of herbs, aiming to enhance therapeutic efficacy, reduce potential toxicity, and improve storage stability (Li et al., 2020; Wu et al., 2018). For *Dendrobium* species, classical manuals like Leigong Paozhi lununderscore the significance of processing. Wine-steaming (*Jiuzheng*), a method involving moistening the herb with yellow rice wine followed by steaming, is regarded as particularly consequential for this genus (Li et al., 2023; Yang et al., 2015). However, despite its established traditional rationale, *D huoshanense* is still predominantly utilized in its raw or simply dried form in contemporary practice. This represents a disconnect from the principle of *Paozhi* and underscores a gap between historical knowledge and modern application (Trifan et al., 2022; Zhu et al., 2023). Crucially, the specific impact of wine-steaming on the yield, structural characteristics, and most importantly, the anti-inflammatory bioactivity of *D huoshanense* polysaccharides (DHP) remains scientifically unclear. Current research on the processing of medicinal plants often focuses on optimizing single parameters for polysaccharide extraction or modification. A critical research gap remains in the systematic and multi-index optimization of complex traditional processes like wine-steaming and in directly linking such optimized processing parameters to subsequent changes in polysaccharide structure and enhanced anti-inflammatory potency.

To address this gap, the present study integrated modern optimization tools with pharmacological evaluation to systematically examine the wine-steaming process of *D huoshanense*. Key processing parameters, namely wine concentration, moistening time, and steaming duration, were optimized using a combined Entropy Weight Method (EWM) and Response Surface Methodology (RSM) approach. EWM is an objective multi-index evaluation method that assigns weights according to the degree of dispersion of each response indicator, thereby reducing subjective bias in comprehensive assessment (Cai et al., 2019; Zhang et al., 2021; Zhu and Liu, 2013). In this study, polysaccharide content, alcohol-soluble extract, and water-soluble extract were selected as the response parameters for EWM analysis, because these indices collectively reflect the major compositional and quality-related changes during processing. Based on the comprehensive evaluation value generated by EWM, RSM was further applied to model the relationships between process variables and response values, evaluate factor interactions, and identify the optimal processing conditions. Polysaccharides were subsequently extracted from both raw and optimally wine-steamed samples, and the changes in their physicochemical properties, including molecular characteristics and monosaccharide composition, were further investigated. Their anti-inflammatory activities were evaluated and compared using an LPS-stimulated RAW264.7 macrophage model. Through this integrated methodology, the study aims to provide a scientific validation for the traditional wine-steaming method, thus offering support for the inheritance and innovation of traditional Chinese medicinal processing technology, and to contribute to the standardized processing and development of polysaccharide-based bioactive ingredients from medicinal plants.

## 2 Materials and methods

### 2.1 Materials and instruments

Three-year-old *D huoshanense* samples were collected from Taipingfan Township, Huoshan County, Anhui Province, China. The dried stems were identified by Professor Nianjun Yu (School of Pharmacy, Anhui University of Chinese Medicine) as *D. huoshanense* C.Z. Tang et S.J. Cheng (Orchidaceae family).

Yellow rice wine (Beijing Tapai Brand; complying with the Chinese national standard GB/T 17946, ethanol content ≥ 52%) was used as the processing adjuvant and diluted to the required concentrations before use. The RAW264.7 murine macrophage cell line was obtained from the Shanghai Cell Bank, Chinese Academy of Sciences. Dulbecco’s Modified Eagle Medium (DMEM, #11995), fetal bovine serum (FBS, #KY-01002), phosphate-buffered saline (PBS) buffer powder, lipopolysaccharide (LPS, from *E. coli* O111:B4), Cell Counting Kit-8 (CCK-8), and the total nitric oxide assay kit were purchased from Solarbio (Beijing, China), Tianjin Kangyuan, Biosharp, Sigma-Aldrich, and Beyotime Biotechnology, respectively. A microplate reader (Thermo Scientific Multiskan FC, United States) was used for absorbance measurements, and quantitative real-time PCR analysis was performed using a real-time PCR system (Bio-Rad CFX96, United States). All other chemicals and solvents were of analytical grade.

### 2.2 Experimental design of EWM

#### 2.2.1 Determination of polysaccharide content

Polysaccharide content was determined according to the 2020 edition of Chinese Pharmacopoeia (Commission, 2020). Briefly, samples were defatted by reflux extraction with 80% ethanol, and the precipitate was collected by centrifugation. Polysaccharides were extracted from the precipitate via a 100 °C water bath with ultrapure water, and crude polysaccharides were obtained through ethanol precipitation and washing. For quantification, polysaccharide sample solution was mixed with anthrone-sulfuric acid reagent, heated in a boiling water bath, and absorbance at 620 nm was measured using a microplate reader. DHP content was calculated based on a standard curve established with anhydrous D-glucose.

#### 2.2.2 Determination of water-soluble and alcohol-soluble extractives

The contents of water-soluble and alcohol-soluble extractives were determined using the hot extraction method described in General Chapter 2201 of the 2020 Chinese Pharmacopoeia. Approximately 4 g of sample was accurately weighed, mixed with 100 mL of solvent (water or ethanol), refluxed for 1 h, cooled, and filtered. An aliquot of the filtrate was evaporated to dryness, dried at 105 °C to constant weight, and the extractive content was calculated on a dried basis.

#### 2.2.3 Determination of index weights by EWM

To verify the reliability of the established optimization model, EWM was employed to determine the final weights of each evaluation index. This approach quantifies the amount of information contained in each indicator by assessing the dispersion of the underlying data and assigns weights according to the principle that greater data variability corresponds to lower entropy, higher information content, and consequently higher weight (Song et al., 2021), The calculation procedure of the EWM consists of the following steps: (1) construction of the initial decision matrix; (2) dimensionless normalization of the indicator values; (3) calculation of the proportion of each sample under each indicator; (4) determination of the entropy value for each indicator; and (5) derivation of the final weight coefficients. The steps are defined in Equations 1–5.

$$x_{ij}=\frac{x_{ij}-\min\left\{x_{ij},\cdots \cdots x_{nj}\right\}}{\max\left\{x_{ij},\cdots \cdots x_{nj}\right\}-\min\left\{x_{ij},\cdots \cdots x_{nj}\right\}}$$ (1)

$$P_{ij}=\frac{x_{ij}}{{\sum_{j=1}^{n}x}_{ij}}$$ (2)

$$e_{j}=-k{\sum_{j=1}^{n}P}_{ij}\ln P_{ij},k=\frac{1}{\ln n}$$ (3)

$$d_{j}=1-e_{j}$$ (4)

$$W_{j}=\frac{d_{j}}{{\sum_{j=1}^{n}d}_{j}}$$ (5)

### 2.3 Process optimization of steamed *D huoshanense* with alcohol

#### 2.3.1 Single-factor investigation

Based on literature regarding wine-steaming processing (Chen et al., 2020; Liu et al., 2023; Yu et al., 2023), we selected soaking time with yellow rice wine, dosage of yellow rice wine, and steaming time as investigation factors. A comprehensive score was calculated for each condition.

Moistening Time: Samples (50 g) were moistened with 50 mL yellow rice wine for 2, 4, 6, 8, and 10 h.

Dosage of Yellow Rice Wine: Samples were treated with wine-water mixtures at wine proportions of 20%, 40%, 60%, and 80% (v/v).

Steaming Time: Samples moistened with 40% yellow rice wine for 8 h were steamed for 2, 4, 6, and 8 h.

#### 2.3.2 Optimization of wine-steaming process using RSM

Based on the results of single-factor experiments, a three-factor, three-level Box-Behnken design was employed using Design-Expert 13 software (version 13, Stat-Ease Inc., Minneapolis, MN, United States) (Meneghini et al., 2014). The independent variables and their corresponding levels are presented in Table 1, with the central point (0 level) set according to the optimal conditions identified in the single-factor tests. To enhance methodological transparency, the comprehensive score (*Y*) used as the response value was calculated based on the following weighted formula derived from the entropy weight method:

$$Y=0.471\times \left(\text{Polysaccharide content}\right)+0.366\times \left(\text{Alcohol}-\text{soluble extractives content}\right)+0.163\times \left(\text{Water}-\text{soluble extractives content}\right)$$

**TABLE 1:** Factors and levels for the response surface design of the wine-steaming process.

Level	A: Dosage of yellow rice wine (%)	B: Moistening time (h)	C: Steaming time (h)
−1	20	6	4
0	40	8	6
1	60	10	8

**FIGURE 1:**
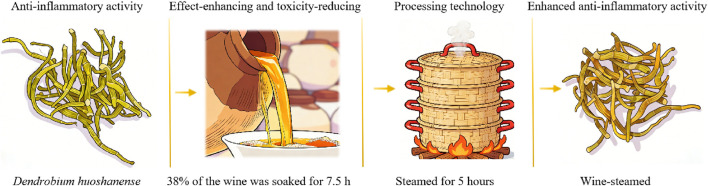
Wine-steaming process flow chart.

Additionally, a schematic diagram of the wine-steaming process (Figure 1) was developed to visually illustrate the key operational steps and parameters.

#### 2.3.3 Validation of the prediction model

Using the optimized parameters predicted by the model (38% yellow rice wine, 7.5 h moistening, 5 h steaming), three batches of *D huoshanense* were processed. The contents of polysaccharides, alcohol-soluble extractives, and water-soluble extractives were determined to validate the model.

### 2.4 Determination of DHP content

Fifty grams of fresh *D huoshanense* were washed, defoliated, and dried to remove leaf sheaths, yielding the raw product of Dendrobium huoshanense. The DHP contents of Dendrobium huoshanense before and after wine-steaming were determined using the method described in “2.2.1”.

### 2.5 Determination of morphological structure

Polysaccharide samples were sieved through a 100-mesh screen. A small amount of the sample was mounted on conductive carbon tape, sputter-coated with a thin layer of gold, and then examined using a field-emission scanning electron microscope (SEM) (Zeiss Sigma 300, Germany). The operational parameters were as follows: SEM mode; acceleration voltage range, 0.02–30 kV; beam current range, 3 pA–20 nA (optionally up to 100 nA); thermal field emission Schottky electron gun with beam stability better than 0.2%/h; and detection using both an in-lens secondary electron detector (in-lens Duo) and an Everhart–Thornley secondary electron detector. The sample chamber dimensions were 365 mm (diameter) × 275 mm (height). Images were acquired at magnifications ranging from ×500 to ×10,000.

### 2.6 Molecular weight determination

To determine the molecular weight distribution and homogeneity of the polysaccharides (Lian et al., 2026), Size Exclusion Chromatography coupled with Multi-Angle Light Scattering and Refractive Index detection (SEC-MALS-RI) was employed. Samples were dissolved in 0.1 M NaNO_3_ containing 0.02% NaN_3_ at 1 mg/mL, filtered through a 0.45 μm membrane, and analyzed on a Thermo U3000 system equipped with a DAWN HELEOS II MALS detector and an Optilab T-rEX RI detector. Separation was carried out on two tandem Ohpak SB-805 HQ and SB-803 HQ columns (300 × 8 mm) at 45 °C, using 0.1 M NaNO_3_ containing 0.02% NaN_3_ as the mobile phase at a flow rate of 0.6 mL/min, with an injection volume of 100 μL and isocratic elution for 75 min. Data acquisition and analysis were performed using ASTRA 6.1 software, and the values of Mw, Mn, and Mw/Mn were calculated with a dn/dc value of 0.141 mL/g.

### 2.7 Determination of monosaccharide composition

The monosaccharide composition of the polysaccharides was determined by HPLC after pre-column derivatization with 1-phenyl-3-methyl-5-pyrazolone (PMP). Briefly, 50 mg of each purified polysaccharide sample was hydrolyzed with 0.5 mL of 3 mol/L HCl in the presence of 1.0 mL of ultrapure water at 116 °C for 1 h in a sealed ampoule. After cooling, the hydrolysate was neutralized with 3 mol/L NaOH and 0.3 mol/L HCl, and diluted to 5 mL with distilled water. An aliquot of 400 μL was mixed with 400 μL of PMP solution and 400 μL of 0.3 mol/L NaOH, and the reaction was carried out at 70 °C for 1.5 h. After derivatization, 500 μL of 0.3 mol/L HCl was added to terminate the reaction. The mixture was extracted with chloroform, and the upper aqueous phase was collected after 2–3 repeated extractions and filtered through a 0.22 μm membrane prior to analysis.

Monosaccharide standards, including mannose (Man), glucuronic acid (GlcA), galacturonic acid (GalA), glucose (Glc), galactose (Gal), and arabinose (Ara), were derivatized using the same procedure as that used for the samples. Calibration curves were established by plotting peak area against the concentration of each standard monosaccharide.

HPLC analysis was performed on a C18 column (100 mm × 2.1 mm, 1.8 μm) with phosphate buffer (pH 6.8) and acetonitrile as the mobile phases. The gradient program was as follows: 0–22 min, 10%–20% acetonitrile; 22–30 min, 20% acetonitrile. The flow rate was 0.2 mL/min, the column temperature was maintained at 30 °C, the detection wavelength was set at 250 nm, and the injection volume was 1 μL. Monosaccharides were identified by comparison of retention times with those of the corresponding standards and quantified using the respective calibration curves.

### 2.8 Comparison of anti-inflammatory activity before and after wine-steaming

#### 2.8.1 Cell culture

RAW264.7 cells were cultured in DMEM complete medium (supplemented with 10% FBS and 1% penicillin-streptomycin) at 37 °C in a humidified atmosphere containing 5% CO_2_ (Deng et al., 2020). Cells were passaged and experiments were conducted using cells between passages 3 and 5.

#### 2.8.2 Cell viability assay (CCK-8)

Cells were seeded in 96-well plates and incubated for 24 h before treatment. RAW264.7 cells were seeded at 5 × 10^4^ cells/well and then treated with DHP (12.5–400 μg/mL) or LPS (1 μg/mL) for 24 h. CCK-8 solution was added, and absorbance at 450 nm was measured after 2 h.

#### 2.8.3 Measurement of nitric oxide (NO) production

Cells were seeded at 4 × 10^5^ cells/well in 6-well plates, treated with DHP (50, 100, 200 μg/mL) or LPS for 24 h, and supernatants were collected. NO concentration was determined using the Griess assay.

#### 2.8.4 Determination of inflammatory cytokines by ELISA

Levels of TNF-α, IL-1β, and IL-6 in cell supernatants were measured using commercial ELISA kits according to the manufacturer’s instructions.

#### 2.8.5 RT-PCR analysis of mRNA expression

Total RNA was extracted using TRIzol reagent, and cDNA was subsequently synthesized according to the manufacturer’s instructions. mRNA expression levels of TNF-α, IL-1β, IL-6, IL-10, iNOS, and COX-2 were quantified by real-time quantitative PCR (RT-qPCR) using the following cycling conditions: 95 °C for 30 s (pre-denaturation), followed by 40 cycles of 95 °C for 5 s (denaturation) and 60 °C for 30 s (annealing). GAPDH was used as the internal reference gene, and primer sequences are listed in Table 2. Relative gene expression was calculated using the 2^−ΔΔCT^ method.

**TABLE 2:** Real-time PCR primer sequences.

Gene	Forward primer	Reverse primer
GAPDH	GGG​TCC​CAG​CTT​AGG​TTC​AT	CCC​AAT​ACG​GCC​AAA​TCC​GT
TNF-α	ACC​CTC​ACA​CTC​ACA​AAC​CAC	ACA​AGG​TAC​AAC​CCA​TCG​GC
IL-1β	GCC​ACC​TTT​TGA​CAG​TGA​TGA​G	TGA​TGT​GCT​GCT​GCG​AGA​TT
IL-6	AGA​CAA​AGC​CAG​AGT​CCT​TCA​G	GTG​ACT​CCA​GCT​TAT​CTC​TTG​GT
IL-10	TTC​TTT​CAA​ACA​AAG​GAC​CAG​C	GCA​ACC​CAA​GTA​ACC​CTT​AAA​G
iNOS	CAA​CAG​GGA​GAA​AGC​GCA​AA	GGG​ATT​CTG​GAA​CAT​TCT​GTG​C
COX-2	GAA​AGC​CCT​CTA​CAG​TGA​CAT​C	GGT​GCT​CCA​AGC​TCT​ACC​AT
β-actin	GTG​CTA​TGT​TGC​TCT​AGA​CTT​CG	ATG​CCA​CAG​GAT​TCC​ATA​CC

### 2.9 Statistical analysis

Data are presented as mean ± SD. Statistical analyses were performed using SPSS 26.0, and GraphPad Prism 8. One-way ANOVA was used for group comparisons. Statistical significance was defined as *p* < 0.05, and extremely significant difference as *p* < 0.01.

## 3 Results and analysis

### 3.1 Calculation of composite weight coefficients by entropy weight method

To improve the interpretability of the comprehensive evaluation, the significance of each selected index was further clarified. Polysaccharide content was included as a key index because polysaccharides are among the major characteristic constituents of the studied material and directly reflect the retention and enrichment of the target active fraction during processing. Alcohol-soluble extractives content was used to characterize the overall changes in alcohol-soluble constituents, thereby providing complementary information on compositional variation induced by different processing conditions. Water-soluble extractives content was included to reflect the level of readily soluble constituents, which is closely related to the practical utilization and overall quality of the processed product. Therefore, these three indices jointly evaluate the sample quality from the perspectives of characteristic active components, overall compositional change, and soluble matter availability.

According to the entropy weight method, the weight coefficients of polysaccharide content, alcohol-soluble extractives content, and water-soluble extractives content were 0.471, 0.366, and 0.163, respectively. These results indicate that polysaccharide content made the greatest contribution to distinguishing the samples under different processing conditions, followed by alcohol-soluble extractives content, whereas water-soluble extractives content contributed relatively less. It should be noted that, in the entropy weight method, a higher weight indicates a greater degree of variation and information contribution among samples, rather than absolute pharmacological importance. Accordingly, the comprehensive evaluation score (*Y*) was calculated as follows:

$$Y=0.471\times \left(\text{Polysaccharide content}\right)+0.366\times \left(\text{Alcohol}-\text{soluble extractives content}\right)+0.163\times \left(\text{Water}-\text{soluble extractives content}\right)$$

### 3.2 Process optimization of steamed *D huoshanense* with alcohol

**FIGURE 2:**
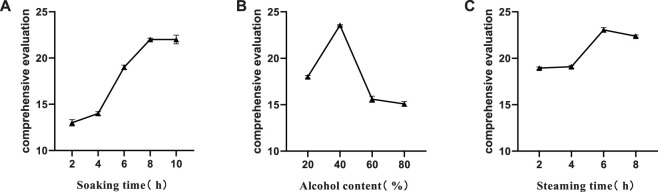
Effects of processing parameters on comprehensive score. **(A)** Moistening time; **(B)** yellow rice wine concentration; **(C)** steaming time. Data are mean ± SD (n ≥ 3).

As shown in Figure 2, the comprehensive score reached its maximum at a moistening time of 8 h, a yellow rice wine concentration of 40%, and a steaming time of 6 h. These parameter levels were therefore selected as the center points (zero levels) for the subsequent response surface optimization. Based on the single-factor results, a three-factor, three-level Box–Behnken design was employed, resulting in 17 experimental runs, and the corresponding results are presented in Table 3.

**TABLE 3:** Response surface method for optimizing the steaming process of *D huoshanense* wine and the results of the experiment.

Test	*A* alcohol content %	*B* soaking time (h)	*C* steaming time (h)	*R1* polysaccharide content (%)	*R2* alcohol soluble extract content %	*R3* water soluble extract content %	*Y* overall score
1	20	6	6	19.26	7.94	39.42	18.4
2	60	6	6	17.56	7.54	37.28	17.11
3	20	10	6	18.3	6.4	40.18	17.51
4	60	10	6	16.06	6.03	41.02	16.46
5	20	8	4	19.37	7.68	41.92	18.77
6	60	8	4	17.98	6.84	41.84	17.79
7	20	8	8	18.84	7.6	39.62	18.11
8	60	8	8	16.17	6.2	34.98	15.59
9	40	6	4	23.53	8.52	43.44	21.28
10	40	10	4	23.21	8.2	42.56	20.87
11	40	6	8	23.43	7.68	43.18	20.88
12	40	10	8	17.02	7.4	40.3	17.29
13	40	8	6	24.28	9.96	43.24	22.13
14	40	8	6	24.28	9.1	41.94	21.6
15	40	8	6	24.07	8.8	41.98	21.4
16	40	8	6	24.07	9.26	43.42	21.8
17	40	8	6	24.28	9.96	43.77	22.22

Regression analysis generated the following quadratic polynomial equation:

$$Y=21.83-0.7314\times A-0.6930\times B-0.8540\times C+0.0606\times AB-0.3878\times AC-0.7949\times BC-3.49\times A^{2}-0.9715\times B^{2}-0.7763\times C^{2}$$

Analysis of variance showed that the model was highly significant (p < 0.01), while the lack of fit was not significant (p = 0.1564), indicating that the model was suitable for describing and optimizing the wine-steaming process of *D huoshanense* (Table 4). Among the three factors, the influence on the comprehensive score ranked as steaming time (C) > wine concentration (A) > moistening time (B). In addition, the interaction between moistening time and steaming time was significant (BC term, p = 0.0118), whereas the AB and AC interaction terms were not significant.

**TABLE 4:** Comprehensive score of variance analysis.

Soruce of variation	Quadratic sum	Variance	Mean square	F-value	P-value
Model	78.44	9	8.72	39.27	<0.0001
A- alcohol content	4.28	1	4.28	19.28	0.0032
B- soaking time	3.84	1	3.84	17.31	0.0042
C- steaming time	5.84	1	5.84	26.29	0.0014
*AB*	0.0147	1	0.0147	0.0662	0.8044
*AC*	0.6015	1	0.6015	2.71	0.1437
*BC*	2.53	1	2.53	11.39	0.0118
*A* ^*2*^	51.26	1	51.26	230.99	<0.0001
*B* ^*2*^	3.97	1	3.97	17.91	0.0039
*C* ^*2*^	2.54	1	2.54	11.43	0.0117
Residual	1.55	7	0.2219	​	​
Lack of fit	1.08	3	0.3594	3.03	0.1564
Pure error	0.4752	4	0.1188	​	​
Gross difference	79.99	16	​	​	​

**FIGURE 3:**
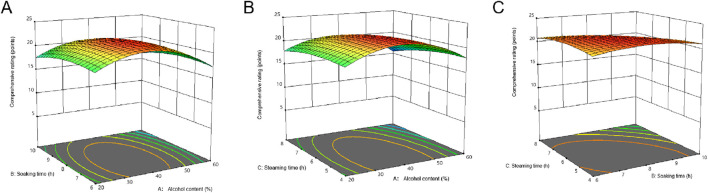
Results of response surface analysis. **(A)** Interaction between liquor content (A) and immersion time (B); **(B)** Interaction between liquor content (A) and steaming time (C); **(C)** Interaction between immersion time (B) and steaming time (C). The color gradient of the surface, from green to red, indicates an increase in the comprehensive score. The density of the contour lines reflects the intensity of the interactive effects of the parameters on the comprehensive score.

The three-dimensional response surface plots further illustrated the interactive effects of the processing variables on the comprehensive score (Figure 3). The model predicted that the optimal processing conditions were 38.4% wine concentration, 7.6 h moistening time, and 5.1 h steaming time, with a predicted comprehensive score of 22.10. Considering practical feasibility and operational convenience, the final optimized conditions were adjusted to 38% wine concentration, 7.5 h moistening time, and 5 h steaming time.

To validate the adequacy of the model, three independent batches were prepared under the optimized conditions. As shown in Table 5, the average comprehensive score was 23.31, with an RSD of 0.50%, indicating good robustness and reproducibility of the optimization model.

**TABLE 5:** Validation results for the optimized wine-steaming process.

Batch	Polysaccharide content (%)	Alcohol-soluble extractives (%)	Water-soluble extractives (%)	Comprehensive score
1	26.41	9.86	44.22	23.26
2	26.62	9.97	44.53	23.45
3	26.30	9.93	44.23	23.23
Average	26.44	9.92	44.33	23.31
Standard deviation	0.16	0.06	0.18	0.12
*RSD*/%	0.61	0.56	0.40	0.50

### 3.3 Determination of DHP content before and after wine-steaming

**FIGURE 4:**
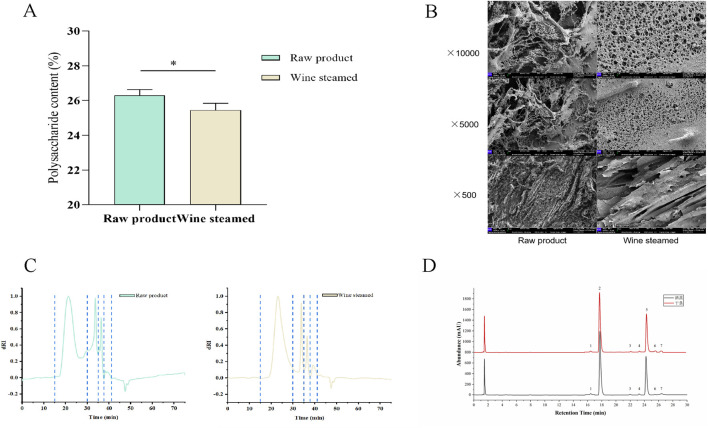
Physicochemical characterization of polysaccharides before and after wine steaming. **(A)** Determination of polysaccharide content before and after wine steaming. Data are presented as mean ± SD (n = 3). **(B)** SEM observation of polysaccharide surface morphology. **(C)** Molecular weight distribution of polysaccharides. **(D)** Monosaccharide composition of polysaccharides.

Ultrapure water (1.0 mL) was used as the blank control, and absorbance was measured at 488 nm. The glucose standard curve was *Y* = 9.7607*X* + 0.0045 (*R* ^*2*^ = 0.9991), demonstrating good linearity within the concentration range of 0.01–0.08 mg/mL. Quantitative results showed that the polysaccharide content of *D. huoshanense* decreased significantly after wine-steaming, from 26.29% ± 0.34% to 25.45% ± 0.40% (*P* < 0.05; Figure 4A), representing a 3.7% reduction. This suggests that wine-steaming affects the material basis of *D. huoshanense* and is associated with a partial loss of polysaccharides.

### 3.4 Morphological observation

Scanning electron microscopy (SEM) was employed to investigate the morphological characteristics of polysaccharides from raw and wine-steamed *D. huoshanense*, revealing evident structural differences between the two samples. As shown in Figure 4B, at a magnification of ×500, the raw polysaccharides exhibited a relatively compact sheet-like and fibrous morphology with a dense surface and limited pores. In contrast, the wine-steamed polysaccharides displayed a more disrupted structure, characterized by fractured lamellae, surface peeling, and a looser overall texture.

tAt a magnification of ×5,000, the raw polysaccharides showed an irregular network with unevenly distributed cavities and filamentous connections. Under the same magnification, the wine-steamed polysaccharides presented a more pronounced porous architecture with a higher number of interconnected pores and a more uniform sponge-like structure. These observations indicated that wine-steaming markedly altered the surface morphology and internal organization of the polysaccharide matrix.

At a higher magnification of ×10,000, the raw polysaccharides appeared as entangled fibrillar and lamellar aggregates with a rough and heterogeneous surface, accompanied by irregular protrusions and scattered particulate attachments. By comparison, the wine-steamed polysaccharides exhibited a more developed porous network with densely distributed micropores and granular features across the surface. The processed polysaccharides displayed a looser and more open microstructure, suggesting that wine-steaming promoted structural rearrangement and partial disruption of the original compact matrix.

Overall, the morphological features of DHP changed significantly after wine-steaming. Compared with the raw sample, the processed polysaccharides exhibited a looser texture, a higher degree of porosity, and a more interconnected network structure. Such structural changes may facilitate the exposure of functional groups and enhance interactions with the surrounding environment, which may be associated with the improved biological activity observed after processing.

### 3.5 Determination of molecular weight

The molecular weight distribution of polysaccharides is closely associated with their physicochemical properties and biological activities. In this study, SEC-MALS-RI was used to characterize the molecular weight distribution of polysaccharides from raw and wine-steamed *D*. *huoshanense*. As shown in Figure 4C, both samples exhibited one predominant polysaccharide fraction. Compared with the raw product, the peak range of the wine-steamed sample shifted from 17.50 to 35.50 min to 19.00–35.50 min, accompanied by a decrease in RI peak area from 0.0000228071 to 0.0000170024 RIU·min.

The raw polysaccharides showed Mn, Mp, Mw, and Mz values of 89.974, 230.302, 240.460, and 488.699 kDa, respectively, with a polydispersity index (Mw/Mn) of 2.673. After wine steaming, Mn, Mp, and Mw decreased to 22.577, 160.302, and 218.484 kDa, respectively, whereas Mz increased markedly to 1,400.202 kDa, and the polydispersity index rose to 9.677. These results indicate that wine steaming substantially altered the molecular weight distribution and decreased the homogeneity of DHP. The marked reduction in Mn and Mp suggests the generation of lower-molecular-weight fractions during processing, whereas the increase in Mz may reflect the presence of a small proportion of high-molecular-weight aggregates or associated macromolecular species. Collectively, these findings suggest that wine steaming promoted molecular redistribution and increased the heterogeneity of DHP.

### 3.6 Monosaccharide composition

To investigate the effect of wine-steaming on the structural characteristics of DHP, the monosaccharide composition of polysaccharides isolated from raw and wine-steamed samples was analyzed. As shown in Table 6, both samples were composed mainly of mannose, glucose, galactose, and arabinose, together with minor amounts of glucuronic acid and galacturonic acid. These results indicate that wine-steaming did not alter the monosaccharide species present in DHP (Figure 4D), but it did affect their relative molar proportions.

**TABLE 6:** Monosaccharide composition of polysaccharides from raw and wine-steamed *D huoshanense*. Values are expressed as mean ± SD (n = 3).

Sample	Wine-steamed product (%)	Raw product (%)
Mannose	42.35 ± 1.14	52.39 ± 0.95
Glucuronic acid	1.17 ± 0.32	1.21 ± 0.12
Galacturonic acid	1.25 ± 0.09	1.81 ± 0.10
Glucose	33.43 ± 1.28	28.74 ± 0.79
Galactose	3.61 ± 0.26	1.73 ± 0.16
Arabinose	4.97 ± 0.22	2.65 ± 0.17

Mannose remained the predominant monosaccharide in both groups; however, its proportion decreased markedly from 52.39% ± 0.95% in the raw product to 42.35% ± 1.14% in the wine-steamed product. In contrast, the glucose content increased from 28.74% ± 0.79% to 33.43% ± 1.28% after wine-steaming. As a result, the mannose-to-glucose ratio decreased from approximately 1.82 in the raw product to 1.27 in the wine-steamed product. In addition, the contents of galactose and arabinose increased from 1.73% ± 0.16% and 2.65% ± 0.17% in the raw product to 3.61% ± 0.26% and 4.97% ± 0.22% in the wine-steamed product, respectively, whereas galacturonic acid decreased from 1.81% ± 0.10% to 1.25% ± 0.09%. By comparison, the glucuronic acid content showed only a slight change between the two groups.

These compositional changes indicate that wine-steaming affected the monosaccharide distribution pattern of DHP. The decrease in mannose together with the increase in glucose, galactose, and arabinose suggests that processing may alter the structural organization of the polysaccharides. Notably, the elevated galactose content, together with the increase in arabinose, may contribute to the improved anti-inflammatory activity of the wine-steamed polysaccharides, since galactose-containing and arabinogalactan-related structural domains are frequently associated with immunomodulatory effects in bioactive polysaccharides (Chen et al., 2022).

### 3.7 Anti-inflammatory activity comparison of DHP before and after wine-steaming

**FIGURE 5:**
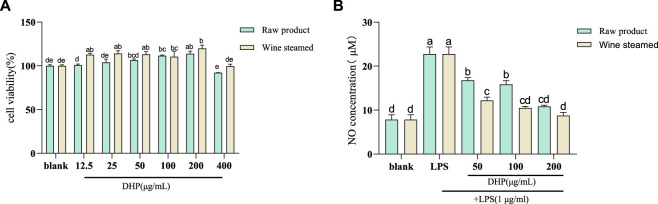
Effects of raw and wine-steamed DHP on cell viability and nitric oxide (NO) production in RAW264.7 macrophages. **(A)** Cell viability of RAW264.7 macrophages treated with raw or wine-steamed DHP at different concentrations (12.5–400 μg/mL); **(B)** NO production in LPS-stimulated RAW264.7 macrophages treated with raw or wine-steamed DHP at different concentrations (50, 100, and 200 μg/mL). RAW264.7 macrophages were pretreated with DHP for 24 h and then stimulated with LPS (1 μg/mL). The blank group served as the untreated control, and the LPS group served as the inflammatory model control. Data are presented as mean ± SD (n = 3). Different letters above the bars indicate significant differences among groups (*P* < 0.05); bars sharing at least one common letter are not significantly different.

Cell viability was assessed to determine the biosafety of DHP in RAW264.7 macrophages. As shown in Figure 5A, treatment with either raw or wine-steamed DHP did not induce cytotoxicity, with all cell viability values remaining above 90%. Both samples increased cell viability to varying extents at 12.5–200 μg/mL, and the wine-steamed DHP generally exhibited a more pronounced stimulatory effect than the raw product. The strongest effect was observed at 200 μg/mL. In contrast, at 400 μg/mL, the cell viability-promoting effect of raw DHP was reduced, while the wine-steamed DHP still maintained a viability level comparable to that of the blank group. Therefore, 12.5–200 μg/mL was selected for the subsequent functional assays.

The role of nitric oxide (NO) in neuroinflammation has been well established in animal models, where it modulates inflammatory processes through key regulatory pathways (Dai et al., 2018). As shown in Figure 5B, LPS stimulation markedly increased NO production in RAW264.7 macrophages compared with the blank group, indicating successful induction of the inflammatory model. Both raw and wine-steamed DHP significantly inhibited LPS-induced NO release at 50–200 μg/mL. Although NO levels generally decreased with increasing DHP concentration, the trend was not strictly concentration-dependent according to the statistical grouping. Compared with raw DHP, wine-steamed DHP exhibited a stronger inhibitory effect at 50 and 100 μg/mL. At 200 μg/mL, both treatments reduced NO production to levels close to that of the blank group, with no obvious difference between the two preparations. These results suggest that wine-steaming enhances the NO-suppressive activity of DHP to some extent.

**FIGURE 6:**
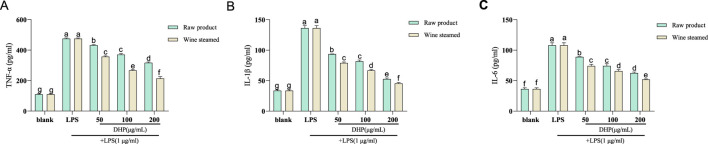
Effects of raw and wine-steamed DHP on TNF-α, IL-1β, and IL-6 secretion in LPS-stimulated RAW264.7 macrophages. **(A)** TNF-α; **(B)** IL-1β; **(C)** IL-6. RAW264.7 macrophages were pretreated with raw or wine-steamed DHP at 50, 100, and 200 μg/mL for 24 h, followed by stimulation with LPS (1 μg/mL). The blank group was used as the untreated control, whereas the LPS group was used as the inflammatory model control. Values are expressed as mean ± SD (n = 3). Different letters indicate statistically significant differences among groups (P < 0.05).

As shown in Figure 6, LPS stimulation markedly increased the secretion of TNF-α, IL-1β, and IL-6 in RAW264.7 macrophages compared with the blank group, confirming successful model establishment. Both raw and wine-steamed DHP significantly inhibited the production of these proinflammatory cytokines at 50–200 μg/mL. Overall, cytokine levels gradually decreased with increasing DHP concentration. Notably, wine-steamed DHP exhibited significantly stronger inhibitory effects than raw DHP at the corresponding concentrations, with the most pronounced suppression observed at 200 μg/mL. These results indicate that wine-steaming enhances the anti-inflammatory activity of DHP in LPS-stimulated RAW264.7 macrophages.

**FIGURE 7:**
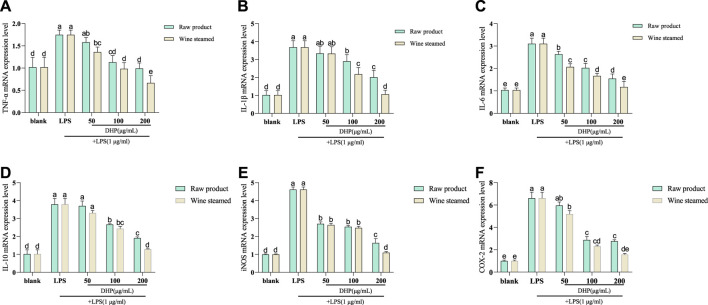
Effects of raw and wine-steamed DHP on the mRNA expression levels of proinflammatory cytokines in LPS-stimulated RAW264.7 macrophages. **(A)** TNF-α mRNA expression level; **(B)** IL-1β mRNA expression level; **(C)** IL-6 mRNA expression level; **(D)** IL-10 mRNA expression level; **(E)** iNOS mRNA expression level; **(F)** COX-2 mRNA expression level. RAW264.7 macrophages were pretreated with raw or wine-steamed DHP (50, 100, and 200 μg/mL) for 24 h and then stimulated with LPS (1 μg/mL). The blank group served as the untreated control, and the LPS group served as the inflammatory model control. Data are presented as mean ± SD (n = 3). Different letters indicate statistically significant differences among groups (P < 0.05).

As shown in Figure 7, LPS markedly increased the mRNA expression of TNF-α, IL-1β, IL-6, IL-10, iNOS, and COX-2 in RAW264.7 macrophages compared with the blank group. Pretreatment with both raw and wine-steamed DHP attenuated these LPS-induced transcriptional changes at 50–200 μg/mL, although the extent of inhibition differed among targets.

For TNF-α, wine-steamed DHP significantly reduced mRNA expression at all tested concentrations, whereas raw DHP showed no significant effect at 50 μg/mL. For IL-1β, neither preparation significantly suppressed expression at 50 μg/mL, but both markedly decreased its expression at 100 and 200 μg/mL, with stronger inhibition observed in the wine-steamed group. For IL-6, both preparations induced a concentration-dependent decrease, and the wine-steamed product consistently produced lower expression levels than the raw product at the corresponding doses.

A similar trend was observed for IL-10, iNOS, and COX-2. Neither raw nor wine-steamed DHP significantly reduced IL-10 expression at 50 μg/mL, whereas both significantly suppressed its expression at 100 and 200 μg/mL, with wine-steamed DHP showing a more pronounced effect at 200 μg/mL. For iNOS, both preparations significantly downregulated mRNA expression relative to the LPS group, and the strongest inhibition was observed at 200 μg/mL, particularly in the wine-steamed group. For COX-2, raw DHP showed no significant inhibition at 50 μg/mL, whereas wine-steamed DHP already produced a significant suppressive effect at this concentration; at 100 and 200 μg/mL, both preparations markedly reduced COX-2 expression, with stronger inhibition in the wine-steamed group at 200 μg/mL.

## 4 Discussion

### 4.1 Optimization of wine-steaming processing conditions

In traditional Chinese medicine, wine-steaming (Jiuzheng) is a classical processing method where yellow rice wine acts as an excipient synergizing with thermal treatment to modify the chemical profile of herbal materials, thereby enhancing their therapeutic efficacy (Chen et al., 2018; Li et al., 2025; Ya-Zhu et al., 2025). The ethanol in wine may function as both a solvent and a penetration enhancer, facilitating the transformation and release of bioactive constituents—a concept aligning with modern pharmaceutical principles of excipient-mediated modulation (Wang et al., 2022), Despite the long-standing application of wine-steaming to Dendrobium species, systematic investigation into its impact on D huoshanense remains limited (Wang, 2021). Particularly concerning its DHP, which represents a key bioactive component with documented antioxidant and anti-inflammatory properties (Ge et al., 2018; Hao et al., 2025).

The EWM was used to determine the weights of the evaluation indicators, which avoided the subjectivity of artificial weighting and made the comprehensive score more objective and reliable (Guo and Tang, 2025; Huang et al., 2021; Qiu et al., 2023). Total polysaccharide content, alcohol-soluble extractive content, and water-soluble extractive content were selected as the core evaluation indices for optimizing the wine-steaming process of *D. huoshanense*, with weights of 0.471, 0.366, and 0.163, respectively. Among them, total polysaccharide content was used as the principal indicator because polysaccharides are the main constituents investigated in this study and are closely associated with the anti-inflammatory activity of *D. huoshanense*. In contrast, alcohol-soluble extractive content and water-soluble extractive content were included as complementary process-evaluation indices to reflect the overall changes in extractable constituents under different wine-steaming conditions. Therefore, these three indices together provide a more comprehensive basis for evaluating processing quality than any single index alone. Based on this evaluation system, RSM with a BBD was applied to optimize yellow rice wine concentration, moistening time, and steaming time. The quadratic regression model was highly significant and showed good predictive performance, and the optimal conditions were determined to be 38% yellow rice wine, 7.5 h moistening time, and 5 h steaming time, thus providing a standardized processing method for *D. huoshanense*. It should be noted that the present study did not further characterize the specific chemical constituents of the alcohol-soluble or water-soluble extractive fractions. Therefore, their contribution to the observed bioactivity should be interpreted as reflecting overall processing-induced compositional changes rather than direct evidence for specific active compounds.

### 4.2 Wine-steaming-induced changes in polysaccharides

Wine-steaming resulted in a slight reduction in the total polysaccharide content of *D. huoshanense*, but significantly enhanced its anti-inflammatory activity, suggesting that bioactivity may be more closely related to structural remodeling than to the absolute polysaccharide yield. In the present study, the total polysaccharide content decreased only modestly from 26.29% ± 0.34% to 25.45% ± 0.40% after wine-steaming, indicating that the overall polysaccharide level was only slightly affected by processing. SEM observations further revealed evident morphological differences between the raw and wine-steamed polysaccharides. The raw polysaccharides exhibited a relatively compact and heterogeneous structure, whereas the wine-steamed polysaccharides showed a looser and more open architecture, characterized by disrupted lamellar structures, increased porosity, and a more interconnected network. These results indicate that wine-steaming markedly altered the microstructural organization of the polysaccharide matrix.

Consistent with the SEM results, molecular weight analysis showed that wine-steaming markedly altered DHP by shifting the polysaccharide peak range to a later retention time, decreasing RI peak area as well as Mn, Mp, and Mw, while markedly increasing Mz and polydispersity, thereby indicating substantial molecular redistribution during processing. These changes indicated that the polysaccharide chains underwent partial depolymerization and molecular redistribution during wine-steaming, resulting in increased low-molecular-weight fractions and reduced homogeneity. The increase in Mz further suggested that a small proportion of high-molecular-weight aggregates or associated macromolecular species might also be formed during processing. These findings are in line with previous reports showing that thermal processing can alter the molecular characteristics of polysaccharides (Bai et al., 2021). More specifically, monosaccharide composition analysis showed marked changes after wine-steaming. Mannose decreased from 52.39% ± 0.95% to 42.35% ± 1.14%, whereas the monosaccharide composition changed markedly, with mannose decreasing from 52.39% ± 0.95% to 42.35% ± 1.14% and glucose increasing from 28.74% ± 0.79% to 33.43% ± 1.28%, such as *Polygonatum sibiricum* and *Cornus officinalis* (Han et al., 2024; Song et al., 2024). As a result, the mannose-to-glucose molar ratio declined from approximately 1.82 to 1.27. These changes suggest that wine-steaming may induce structural reorganization of DHP, including partial degradation or rearrangement of mannose-rich regions and relative enrichment of glucose-containing domains (Zhao et al., 2023). The monosaccharide composition analysis showed that the mannose-to-glucose molar ratio decreased after wine-steaming, indicating that the polysaccharide structure might undergo depolymerization or rearrangement, consistent with previous findings that processing can alter polysaccharide structural features including monosaccharide composition and molecular weight distribution (Guan et al., 2026; Wang et al., 2024; Zhu et al., 2025).

### 4.3 Anti-inflammatory activity of raw vs. wine-steamed DHP

The LPS-stimulated RAW264.7 macrophage model is a classic *in vitro* model for evaluating anti-inflammatory activity (Weiss and Schaible, 2015). Macrophages play a key role in the inflammatory response by secreting NO and pro-inflammatory cytokines such as TNF-α, IL-1β, and IL-6 (Chen et al., 2023). n the present study, processed DHP significantly inhibited LPS-induced NO production and markedly downregulated the mRNA and protein expression levels of TNF-α, IL-1β, IL-6, IL-10, iNOS, and COX-2. These results indicate that processed DHP exerts its anti-inflammatory effects by modulating macrophage activation at both transcriptional and translational levels. These findings provide experimental evidence supporting the traditional use of wine-steamed *D huoshanense* in the treatment of inflammatory conditions and offer a scientific rationale for its clinical application. Notably, the observation that reduced polysaccharide content is accompanied by enhanced anti-inflammatory efficacy is not uncommon in herbal processing. This phenomenon highlights a core principle of Paozhi, whereby therapeutic activity is often optimized through qualitative structural modifications of bioactive constituents rather than through simple quantitative preservation (Gu et al., 2013; Li et al., 2024; Yin et al., 2021). Similar trends have been reported for other processed medicinal herbs, such as Polygonatum sibiricum (Su et al., 2023) and wine-steamed Rheum palmatum (Song et al., 2024), both of which exhibit decreased polysaccharide levels after processing while displaying enhanced anti-inflammatory activity.

In the present study, wine-steaming slightly reduced the total polysaccharide content of *D. huoshanense*, but markedly altered its monosaccharide composition, particularly by decreasing the proportion of mannose and increasing that of glucose. Accordingly, the glucose-to-mannose ratio increased after processing, suggesting that wine-steaming induced structural remodeling rather than a simple quantitative loss of polysaccharides. This compositional shift may be biologically relevant because the immunomodulatory activity of plant polysaccharides is highly dependent on monosaccharide composition, glycosidic linkage patterns, molecular weight distribution, and higher-order conformation. In particular, a higher proportion of glucose residues may change the spatial exposure and receptor-recognition properties of glucose-containing polysaccharide domains, thereby altering their interaction with macrophage pattern-recognition receptors and downstream inflammatory signaling pathways. Under inflammatory conditions, such structural remodeling may enhance the ability of DHP to regulate macrophage overactivation and suppress excessive production of NO and pro-inflammatory cytokines. Therefore, the enhanced anti-inflammatory activity observed after wine-steaming may be associated not only with overall polysaccharide transformation, but also with the relative enrichment of glucose-containing structural motifs. The consistency of these observations across different medicinal plant species further supports the scientific validity of wine-steaming as an effective strategy for optimizing the pharmacological properties of polysaccharide-rich herbal medicines.

### 4.4 Limitations of the study

Although wine-steaming was shown to alter the monosaccharide composition, molecular characteristics, and anti-inflammatory activity of DHP, the precise mechanism by which these structural changes, particularly the increased glucose proportion, regulate macrophage inflammatory responses remains unclear. More detailed structural characterization, including glycosidic linkage patterns, branching features, higher-order conformation, and molecular weight distribution, together with receptor-level mechanistic studies, will be required to clarify the underlying structure–activity relationship. In addition, whether wine-steaming induces conserved structural remodeling patterns in polysaccharides from other medicinal plants has yet to be determined. Future studies should therefore extend this strategy to a broader range of polysaccharide-rich herbal medicines, with the aim of identifying common processing-induced structural signatures and establishing more general structure–activity relationship principles to support the standardized processing and functional development of traditional Chinese medicinal materials.

## 5 Conclusion

This study employed an integrated RSM–EWM optimization strategy to determine the optimal wine-steaming conditions for *D huoshanense* (38% yellow rice wine, 7.5 h of moistening, and 5 h of steaming). Although the polysaccharide yield decreased slightly after processing, the anti-inflammatory activity of the wine-steamed polysaccharides was markedly enhanced, as evidenced by stronger inhibition of nitric oxide production and significant downregulation of key pro-inflammatory mediators. These results indicate that wine-steaming can enhance polysaccharide bioactivity through structural modification, providing both theoretical and experimental support for the standardized processing of *D huoshanense* and the development of anti-inflammatory polysaccharide-based therapeutics.

## Data Availability

The original contributions presented in the study are included in the article/supplementary material, further inquiries can be directed to the corresponding authors.
